# The necessity of treating asymptomatic bacteriuria with antibiotics in the perioperative period of joint arthroplasty: a metaanalysis.

**DOI:** 10.3906/sag-2003-22

**Published:** 2021-04-30

**Authors:** Sayed Abdulla JAMI, Jiandang SHI, Zhanwen ZHOU, Changhao LIU

**Affiliations:** 1 Department of Spinal Surgery, Faculty of Surgery, Ningxia Medical University, Yinchuan China

**Keywords:** Asymptomatic bacteriuria, antibiotic, arthroplasty, metaanalysis

## Abstract

**Background/aim:**

Oral antibiotics are usually used to treat asymptomatic bacteriuria during the perioperative period of joint replacement. However, there is no unified conclusion as to whether asymptomatic bacteriuria causes infection around joint prostheses, and the efficacy of antibiotics is unknown.

**Materials and methods:**

We systematically searched PubMed, CNKI, Ovid, Cochrane Library, EMBASE, manual research, and references of relevant articles up to January 1, 2020, to identify and compare observational studies. The Cochrane systematic review method was used, and Review Manager 5.3 software was used for analysis.

**Results:**

Nine articles were included in the analysis, involving 29,844 cases of joint arthroplasty and 2366 cases of asymptomatic bacteriuria. Periprosthetic joint infection had a significantly higher incidence in the asymptomatic bacteriuria group than in the nonasymptomatic bacteriuria group (Odds Ratio: OR = 3.15, 95% CI: 1.23–8.02, P = 0.02). Seven of the nine articles reported the use of antibiotics for treating perioperative asymptomatic bacteriuria and there was no significant difference in the incidence of periprosthetic joint infection between the two groups (OR = 1.64, 95% CI: 0.84–3.23, P = 0.15).

**Conclusion:**

The occurrence of asymptomatic bacteriuria in the perioperative period of joint arthroplasty is a risk factor for periprosthetic joint infection, and the use of antibiotics for asymptomatic bacteriuria does not change the rate of incidence.

## 1. Introduction 

Periprosthetic infection is a fatal complication of joint replacement surgery, with an incidence of 1% to 2% for primary joint replacement and 3% to 5% for resurgery [1,2]. Even with adequate preoperative preparation and antibiotic prevention, the occurrence of joint periprosthetic infections is unavoidable. As the amount of arthroplasty continues to increase,the economic burden of prosthetic joint infections in the aging population is increasing significantly [3]. Therefore, it is crucial to identify preoperative risk factors for timely prevention, early diagnosis, and reasonable treatment.

Asymptomatic bacteriuria is a type of stale urinary tract infection in which the patient has bacteriuria (two successive cultures of clean midstream bacteria greater than 108 L-1 and the same bacteria species twice in a row, with the specific exclusion, not positives) without any symptoms of urinary tract infection. Asymptomatic bacteriuria may be transmitted through blood sources leading to this infection. Although it may be theoretical, it is enough to cause concern among orthopedic surgeons. Patients may develop acute symptomatic urinary tract infections intermittently during the long course of the disease. Studies have shown that urinary tract infection may cause a hematogenous infection of artificial hip and knee joints [4–6]. However, whether asymptomatic bacteriuria can cause artificial joint infection remains to be confirmed. Some scholars reported that asymptomatic bacteriuria did not affect the incidence of periarticular infection after joint replacement, and even if bacterial infections around periprosthetic infections are present, they are different from urinary tract infections [7–9]. Others reported that asymptomatic bacteriuria increased the incidence of postoperative periarticular prosthesis infection but only in retrospective studies where the quality of evidence was low [10,11]. At present, different guidelines are also contradictory: The British Orthopaedic Association guidelines [12] support routine preoperative urine screening, but do not indicate whether treatment is required; the Scottish Intercollegiate Guidelines Network [13] states that patients should not be treated with antibiotics for asymptomatic bacteriuria unless pregnant. Whether perioperative antibiotic treatment of asymptomatic bacteriuria can reduce the risk of subsequent joint periprosthetic infections is also a huge clinical controversy; at present, many clinical physicians use empirical antibiotics for asymptomatic bacteriuria patients and delay the operation time, which may lead to the overuse of antibiotics and waste of resources. Therefore, this study collected and sorted the previously published literature and a metaanalysis was conducted for correct evaluation of: (1) The effect of asymptomatic bacteriuria in the perioperative period of joint replacement on periarticular infection of a prosthesis; (2) The effect of perioperative asymptomatic bacteriuria antibiotic treatment on the periprosthetic joint infection.

## 2. Methods and materials

### 2.1. Literature selection criteria

Inclusion criteria: 1. Cases of joint replacement included total knee replacement, total hip replacement, and hemiarthroplasty; 2. The literature was divided into experimental group and control group based on asymptomatic bacteriuria; 3. The data provided can be used to assess the relative risk (OR); 4. Prognostic results include prosthetic periarticular infection.

Exclusion criteria: 1. If other infections of asymptomatic bacteriuria or non-prosthesis periarticular complicated infections; 2. If the literature data is incomplete and cannot be extracted; 3. If there are repeated publications; 4. If the article format is review, systematic review, or case report; 5. Literature written in languages other than Chinese or English.

### 2.2. Literature selection

The system searches major foreign databases, such as PubMed, CNKI, Baidu, Ovid, Google Scholar, Cochrane Library, Research Gate, and EMBASE, etc. from 05.30.2018. The keywords used in the search were asymptomatic bacteriuria, pyuria, arthroplasty, hemiarthroplast, prosthetic joint infection, and prosthesis related infections.

Manually selected major orthopaedic journals and recent review references were reviewed by colleagues who were asked to report the results of the completed but unpublished literature and related experimental results of the unfinished experiments, in order to expand the search of the references included in the original literature. Literature screening was performed independently by two authors. In the case where screening results were inconsistent, the third author decided the outcome of the screening results and contacts as necessary.

### 2.3. Literature screening

Data extraction combined all the search results and removed the nonconforming literature according to the title, abstract, and full text. Extracted data included: 1. Basic information: first author, year of publication, research area; 2. Literature eligibility data: study type, sample size, general demographic data, the sample size of each study group, joint replacement ratio, whether the pathogenic bacteria in urine culture are consistent with the bacteria around the joint prosthesis, antibiotic use of each study group (including use of time, use of drugs); 3. Methodological data: literature research type. All data were crosschecked, and the third author determined any inconsistencies.

### 2.4. Quality evaluation

The quality of the included studies was assessed using the Newcastle–Ottawa scale (NOS) [14]. Using the NOS tool for each study is judged on eight items, categorized into three groups: the selection of the study groups; the comparability of the groups; and the ascertainment of either the exposure or outcome of interest for case-control or cohort studies, respectively. The NOS’s evaluation of document quality uses the semiquantitative principle of a star system [15]. The full score is 9 stars (☆). The literature review, inclusion, and assessment of the literature were carried out independently by two reviewers. If there were differences in the evaluation results, these were solved through discussion or third-party consultation. If necessary, contact the original author for further clarification.

### 2.5. Statistical methods and metaanalysis

The observed selected data of each document were extracted and recorded using the Review Manager 5.3 software for metaanalysis by two independent reviewers. The OR was selected as the effective size for continuous variables, and Review Manager 5.3 was used to calculate I2 and P-value to test for heterogeneity, P > 0.1 or I2 < 50%, the heterogeneity among the studies was considered to be small, and a fixed outcome model was used to combine outcome variables. If P < 0.1 or I2 > 50%, the heterogeneity among the studies was considered to be relatively large. After excluding the clinical heterogeneity and statistical heterogeneity of the literature, the outcome variables were combined with the random effect model, and subgroup analysis or sensitivity analysis was performed if necessary. Descriptive analysis was used for data that could not be combined. The degree of heterogeneity of low, medium, and high for I2 statistics were indicated as ≤ 25%, 25% – 50%, ≥ 75%, respectively [16]. The final combined results were represented by forest plot, the Z test tested the total effect values, and P < 0.05 was considered as a significant difference.

## 3. Results 

### 3.1. Literature search results

**Figure 1:**
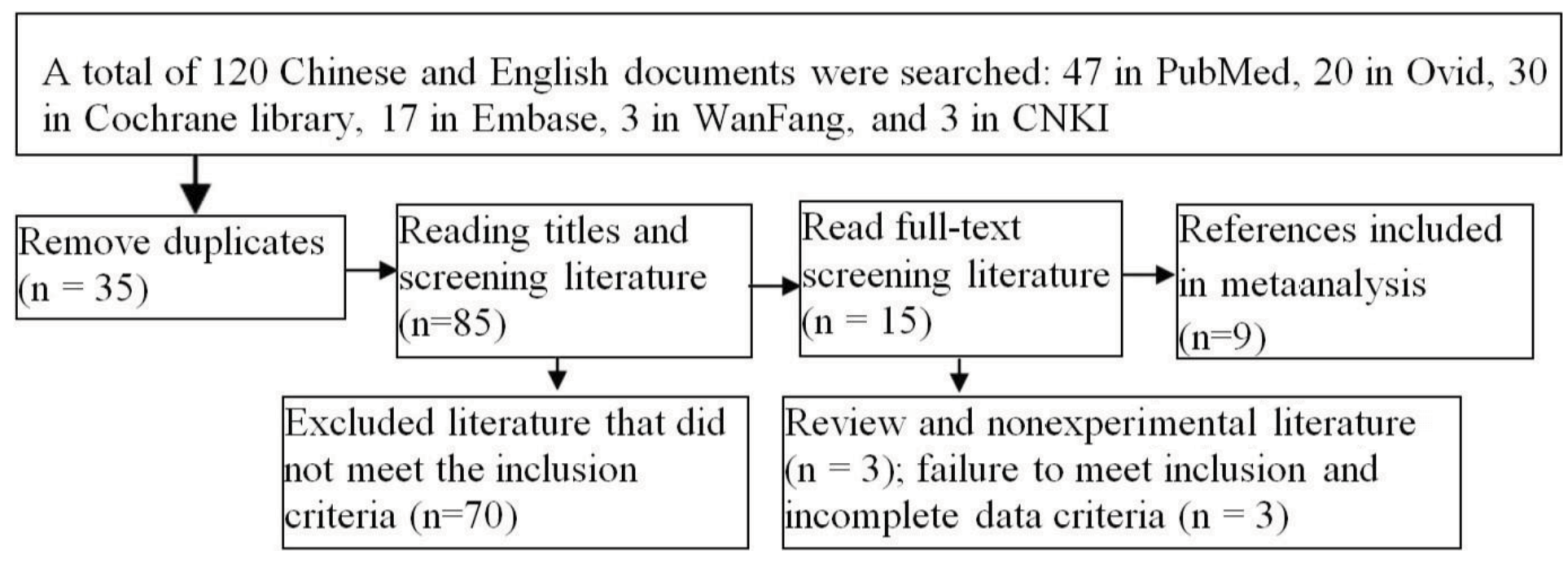
Flow chart of the literature screening.

A total of 120 articles were selected in the first instance, including 47 PubMed articles, 20 Ovid articles, 30 Cochrane library articles, 17 EMBASE articles, 3 WanFang articles, and 3 CNKI articles. No literature was found through manual search, 35 duplicated articles were removed, and 70 articles that did not meet the inclusion criteria were also removed. A total of 15 articles were read in detail, 6 were removed, and the remaining 9 were included [8–11, 17–21]. There were 29,844 cases of joint replacement and 2366 cases of asymptomatic bacteriuria, among which 6 were retrospective cohort studies, 1 was a prospective cohort study, and 2 were prospective randomized controlled studies, as shown in the literature screening process Figure 1. The necessary information about the 9 articles is shown in Table 1. Seven of the nine articles analyzed the effect of the antibiotic application on the prognosis of asymptomatic bacteriuria during the perioperative period of joint replacement Table 2.

**Table 1:** Basic characteristic of the included studies.

Author	Published year	Region	Study design	Sample size (n)	Age(years)	Sex(M/F)	Asymptomatic bacteriuria group/control group (n)	Joint arthroplasty ratio(knee/hip	Follow-up time(month)	Is the urine the same as the bacteria around the joint prosthesis?
Sousa [21]	2014	Portugal	RC study	2497	68	925/1572	303/2196	1249/1248	≥12	Different
Martínez-Vélez[19]	2016	Portugal	PRC study	215	73.4 ± 6.7	47/168	11/204	215/0	48	Different
Honkanen [9]	2017	Finland	RC study	23171	67	8 810/14361	1378/18848	12971/10200	12	Different
Cordero-Ampuero [18]	2013	Portugal	PRC study	471	NA	178/293	46/425	0/471	1-20	Different
Wang [10]	2017	China	RC study	982	56±6	301/681	139/843	559/423	≥12	Different
Weale [11]	2018	England	RC study	5542	68	2214/3328	140/4228	2776/2667	12	1 same, 6 different
Ritter [20]	1987	USA	RC study	277	NA	NA	32/242	97/267	≥12	Different
Glynn [8]	1984	Ireland	RC study	299	NA	NA	57/242	NA	3	Different
Bouvet [17]	2014	Switzerland	RC study	510	69.1	209/309	260/250	220/290	≥12	Different

RC = Retrospective cohort, PRC = Prospective randomized controlled study, NA = Not Available.

**Table 2:** Basic characteristics of the use of antibiotics in the included studies.

Author	Antibiotic treatment /untreated (n)	Duration of antibiotic treatment (d)	Antibiotic selection	Review of urine culture
Sousa [21]	154/149	8 d	UC & drug sensitivity result	NO
Martínez-Vélez [19]	4/7	7 d	UC & drug sensitivity result	NO
Honkanen [9]	344/1085	NA	Effective antibiotic therapy	NA
Cordero-Ampuero [18]	228/243	7 d	UC & drug sensitivity result	NA
Wang [10]	139/843	Preoperative NA/ postoperative 3 day	Random cephalosporins generation 1 & 2	NA
Glynn [8]	18/39	10 d	UC & drug sensitivity result	NA
Bouvet [17]	260/250	5 d	UC & drug sensitivity result	Day 3, after surgery

NA = Not Available, UC = Urine culture.

### 3.2. Methodological quality evaluation of included studies

The NOS was used to evaluate the quality of the included studies, and the evaluation results ranged from 5 to 9 stars, as shown in the results Table 3.

**Table 3:** NOS scores in the included studies.

Author	People selection	Comparability	Exposure or result	Total score
Sousa [21]	☆☆☆	☆☆	☆☆	7
Martínez-Vélez [19]	☆☆☆	☆☆	☆☆☆	8
Honkanen [9]	☆☆	☆	☆☆	6
Cordero-Ampuero [18]	☆☆☆	☆☆	☆☆☆	8
Wang [10]	☆☆☆	☆	☆☆	6
Weale [11]	☆☆☆	☆	☆☆	6
Ritter [20]	☆☆☆	☆	☆☆☆	7
Glynn [8]	☆☆	☆	☆☆	5
Bouvet [17]	☆☆☆☆	☆☆	☆☆☆	9

### 3.3. Results of metaanalysis

**Figure 2:**
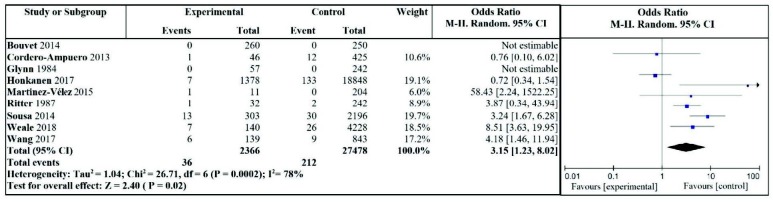
Effect of asymptomatic bacteriuria on the incidence of periprosthetic joint infection.

**Figure 3:**
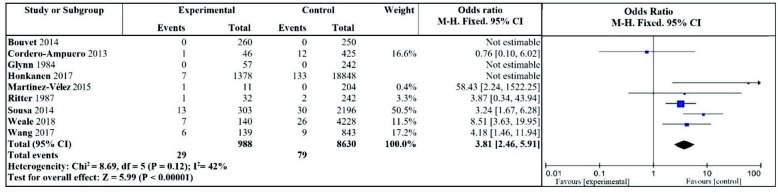
Effect of asymptomatic bacteriuria on the incidence of periprosthetic joint infection (after removal of Honkanen et al. [9] literature).

The relationship between asymptomatic bacteriuria during the perioperative period of joint replacement and postoperative periprosthetic infection was included in 9 studies [8–11, 17–21]. All outcomes included the statistics of postoperative infection rate around the joint prosthesis, and the results of heterogeneity test were: I2 = 78%, P = 0.0002. It was suggested that there was a high degree of heterogeneity in the included studies, thus the random effect model was used to merge the statistics. The results showed that compared with the control group, patients with asymptomatic bacteriuria during the perioperative period of joint replacement were more likely to suffer from periprosthetic infection with a significant difference (OR = 3.15, 95% CI: 1.23–8.02, P = 0.02), as shown in Figure 2 **.** After removing the article by Honkanen et al. [9], heterogeneity decreased: I2 = 42%, P = 0.12, there was moderate heterogeneity, which was demonstrated by the fixed effect model; the difference between the two groups was still significant (OR = 3.81, 95% CI: 2.46–5.91, P < 0.00001). See Figure 3 for the forest plot.

**Figure 4:**
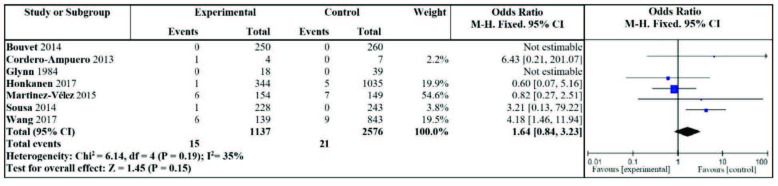
Effect of antibiotic treatment of asymptomatic bacteriuria on the incidence of periprosthetic joint infection.

The relationship between asymptomatic bacteriuria treated with antibiotics during the perioperative period of joint replacement and postoperative periarticular prosthetic infection was included in 7 studies [8–10,17–19,21]. Heterogeneity analysis: I2 = 35%, P = 0.19, there was low heterogeneity, and metaanalysis with a fixed-effect model results show that there is no significant difference in the incidence of periprosthetic joint infections between the nonantibiotic treatment group and the antibiotic treatment group. The antibiotic treatment of asymptomatic bacteriuria during the perioperative period of joint replacement does not reduce the incidence of postoperative periarticular infections (OR = 1.64, 95% CI: 0.84–3.23, P = 0.15) forest graph is shown in Figure 4.

### 3.4. Sensitivity analysis and publication bias

In the sensitivity analysis for the main indicators, different effect models (fixed effects and random effects models) were used for those studies whose selection bias was unclear due to the removal of the random sequence generation method. Regardless of the application of intentionality analysis, the conclusion of the final data combination did not change. The results are stable. Less than ten papers were included in each analysis, so there were no conditions for evaluating publication bias.

## 4. Discussion

Artificial joint infection is one of the most severe complications of joint replacement, bringing high costs to society, families, and individuals. Therefore, prevention of infection around the joint prosthesis is significant for the prognosis of the artificial joint replacement. Previous literature holds different views on whether asymptomatic bacteriuria during the perioperative period can cause periarticular prosthesis infection. For example, a mail survey report in the UK stated that [18] 2/3 of orthopedic surgeons treated asymptomatic bacteriuria before total knee replacement, but 70% of doctors believe that there is no evidence that asymptomatic bacteriuria should be treated before surgery. Sousa et al. [21] conducted a multivariate analysis of 2497 patients with joint replacements and known risk factors for infections. It showed that patients with asymptomatic bacteriuria had 3 times the risk of infection around the joint prosthesis compared with patients with normal urine. In the asymptomatic bacteriuria group, there was no statistically significant difference in the rate of infection around the joint prosthesis between the antibiotic-treated group (3.9%) and the untreated group (4.7%). Therefore, preoperative antibiotic treatment of asymptomatic bacteriuria shows no benefit and is not recommended. It is worth noting that the pathogen cultured around the joint prosthesis is different from the pathogens found in the urine culture of asymptomatic bacteriuria before surgery. This indicates that asymptomatic bacteriuria is not a direct cause of infection around the joint prosthesis. Besides, in a recent study [18], 471 patients were randomly divided into the group that received systemic antibiotics before surgery for 7 days, and the group that did not receive systemic antibiotics before surgery. No pathogens were found to be the same as those found in the culture of asymptomatic bacteriuria and urine after total hip replacement surgery. According to the calculation and analysis to prevent infection around the joint prosthesis, 25,000 patients with asymptomatic bacteriuria need to be treated before surgery.

In this study, 9 literatures were included to analyze the effect of asymptomatic bacteriuria during the perioperative period on postoperative periarticular prosthesis infection. Results suggest that perioperative asymptomatic bacteriuria is a risk factor for postoperative joint periprosthetic infection, and the results were highly heterogeneous. A sensitivity analysis was performed, and it was found that after removing the research data by Honkanen et al. [9], the heterogeneity (I2 = 42%) decreased significantly, and the results were more representative, as shown in Figure 3. In the analysis of the causes, the prevalence of asymptomatic bacteriuria and periarticular prosthesis infection was 6.8% and 0.68%, respectively. The diagnosis of asymptomatic bacteriuria is not mentioned in the materials and methods: firstly, no distinction was made between symptomatic urinary tract infection and asymptomatic bacteriuria, retrospective analysis of all cases from urine culture results, and unbound clinical symptoms and this may lead to the inclusion of a significant number of cases with signs of urinary tract irritation; so there are considerable measurement bias and hybrid deviation; secondly, the diagnosis processes of superficial tissue, deep tissue, and periarticular prosthesis infection were not mentioned. The diagnostic criteria for each infection complication are unclear, leading to the inclusion of some superficial and deep tissue infections including cases of joint prosthetic infections, there may be some measurement bias. Finally, there is a large rate of lost follow-up (up to 12.7%), and there is a significant loss of follow-up bias.

In summary, this paper is excluded from the statistical results. The evidence linking prosthetic joint infection to postoperative urinary tract infection is abundant. The results of this metaanalysis suggested that asymptomatic bacteriuria during the perioperative period was a risk factor for periarticular prosthesis infection, increasing the prevalence of infection around the joint prosthesis; however, in the results of urine culture and joint prosthesis infection culture analysis only 1 case was identical (Weale et al. [11] all cultured Escherichia coli), and it is suggested that the bacteria-infected around the joint prosthesis may not be a urinary source. The inconsistent comparison of pathogenic microorganisms between asymptomatic bacteriuria and periprosthetic joint infections in this study may explain that patients with asymptomatic bacteriuria may be at risk of recurrence of multiple different microorganisms. As for the reason why asymptomatic bacteriuria increases the prevalence of infection around the joint prosthesis, David et al. [22] suggested that this might be related to the decreased immune function of patients in this group, or the increased susceptibility to infection caused by colonized bacteria rather than a direct seeding infection. The antibiotic application may cause resistance to colonized microorganisms, or patients with asymptomatic bacteriuria have other risk factors currently recognized that are closely related to joint periprosthetic infection. Many kinds of literature also reported that the incidence of asymptomatic bacteriuria increased the probability of incision for surface infection [8–9,23, 24], and the specific reasons were also unknown. Weale et al. [11] found that gram-positive bacteria caused joint infection around the prosthesis (89%). Methicillin-sensitive staphylococci were found in knee infections and coagulase-negative in hip infections. Reducing the risk of infection around joint prosthesis interventions should focus on reducing the impact of these engraftment bacteria on the skin surface rather than the gram-negative bacteria in the urine.

The overall incidence of asymptomatic bacteriuria in this article is 6.81%. In patients with joint replacement, the incidence of asymptomatic bacteriuria reaches from 4% to 19% [5,8,18,22,25] and is similar to the results in this article. In a multicenter series of joint replacement literature reports [18], the incidence of sterile bacteriuria was 16.3% in women and 5.0% in men. The number of patients with asymptomatic bacteriuria before joint replacement is significant. Traditionally, multiple midcourse urinary colonies count greater than 108 L-1 are considered infectious, and less than 107 L-1 may be contaminated, a value that is only meaningful in cases of acute urinary tract infections and in cases where antibiotics have not been used. The bacterial count, 1 × 108 L-1 which is lower than traditional indicator may still cause urinary tract infection, in the presence of irritant symptoms a count greater than 1×106 L-1 is also considered a urinary tract infection and is more complicated in patients with long-term antibiotic therapy and immune dysfunction. Preoperative pyuria is a reliable standard, but there is some nonspecificity. In the samples with a bacterial count of less than 1×107 L-1, urine gram staining has been proved to be unable to detect urine bacteria well, and some articles reported that the detection rate was only 20%–30% [26]. This article also uses metaanalysis to conclude that the use of antibiotics had no significant effect on the prevention of periprosthetic joint infection caused by asymptomatic bacteriuria, which was consistent with the current treatment attitude of the medical community towards asymptomatic bacteriuria [27,28]. Conventional urinary tract infections are also sensitive to cephalosporins and penicillin, so preoperative antibiotic prophylaxis can also be used to prevent urinary tract infections. According to Ollivere et al. [24], although all patients with asymptomatic bacteriuria before surgery were treated with effective antibiotics, the incidence of nonhealing incision and superficial infection was still significantly higher than that of patients with sterile urine, so the effectiveness of this measure could not be proved.

Furthermore, it is noted that only a few studies have evaluated the therapeutic effect after the use of antibiotics, including urine culture and routine examination, etc. because it is difficult to judge the therapeutic effect of antibiotics in other articles [17,21]. Conventional joint replacements require prophylactic doses of antibiotics. However, it is still controversial whether patients with asymptomatic bacteriuria need to receive a therapeutic dose of antibiotics before surgery. The use of antibiotics included in this article is the therapeutic dose, usually cephalosporins, and the course of treatment is about one week, as shown in Table 2. Clinically, during the perioperative period of joint replacement surgery, no treatment is required for asymptomatic bacteriuria, which can reduce the use of antibiotics and avoid the abuse of antibiotics. It has specific clinical significance. This article also has limitations. Two out of the 9 articles included were prospective randomized controlled trials, but all were small-scale studies; 6 were retrospective cohort studies, and 1 was a prospective cohort study. Most of the retrospective studies were not randomized, failure to follow the blind method and the lack of a correct random grouping method may lead to selection bias and imbalance in baselines between groups.

The quality of the retrospective cohort study is low, which leads to a low level of evidence in the metaanalysis and may affect the results. The study found that asymptomatic bacteriuria was around the joint prosthesis infection risk factor, but the pathogenic bacteria of asymptomatic bacteriuria and joint prosthesis infection of pathogenic bacteria can differ. It is unclear whether there is concrete reason and logic behind the authors speculating that the merger of asymptomatic bacteriuria in patients with the body’s immune balance has been destroyed and easy to merge with other pathogen infections. Some pathogens in asymptomatic bacteriuria and periarticular prosthesis infection have not been cultured due to limited culture techniques, more well-designed high-quality studies are needed to address this scientific topic. Finally, although 1 article was excluded, there was still moderate heterogeneity in the report. This also reflected that there was some unreliability in the combination of the analysis results, but there were still some limitations that should be interpreted with caution.

##  5. Conclusion 

Through a metaanalysis of the included literature, we concluded that the occurrence of asymptomatic bacteriuria during the perioperative period was a risk factor for periarticular infection of the prosthesis, while the use of antibiotics did not change the incidence of periarticular infection. Multicenter prospective studies are still needed to complement the conclusions.

## Contribution of authors

SAJ, SJ and ZZ designed and implemented the project, collected and sorted out the data, wrote the paper and were responsible for the paper. SAJ, LCH & ZZ carried out project implementation, evaluation, and data collection. SJ and SAJ were responsible for quality control and calibration.

## Informed consent

Review board from Ningxia Medical University Hospital and Academic international students authority reviewed this project manuscript and approved it for publication.

## Financial support

All authors declare that financial support does not affect the views of the article and the data on the statistical analysis of objective results and their reports. No current funding.

## Guidelines for writing

This study complies with the PRISMA guidelines for systematic reviews and metaanalysis reporting.

## Biostatistics

The statistical methods in this paper have been reviewed by biostatistics experts in the first affiliated hospital of Ningxia Medical University.

## Ethics approval

Review board of university and hospital approval was obtained before starting the research.

## Availability of data and material

On request a portion of the data will be available, but not all data are publicly available, due to patient confidentiality.
